# Implementation of a fetal ultrasound telemedicine service: women’s views and family costs

**DOI:** 10.1186/s12884-020-03532-4

**Published:** 2021-01-08

**Authors:** V. J. Smith, A. Marshall, M.L.S Lie, E. Bidmead, B. Beckwith, E. Van Oudgaarden, S. C. Robson

**Affiliations:** 1 Northumbria University, G206, Coach Lane Campus, Newcastle upon Tyne, NE7 7XA UK; 2 The Newcastle upon Tyne Hospitals NHS Foundation Trust, Newcastle upon Tyne, UK; 3 University of Cumbria, Carlisle, Cumbria, UK; 4 Newcastle University, Newcastle upon Tyne, UK; 5 North Cumbria University Hospitals NHS Trust, Cumbria, UK

**Keywords:** Ultrasound, Pregnancy, Fetal medicine, Telemedicine

## Abstract

**Background:**

The complexity of fetal medicine (FM) referrals that can be managed within obstetric units is dependent on the availability of specialist ultrasound expertise. Telemedicine can effectively transfer real-time ultrasound images via video-conferencing. We report the successful introduction of a fetal ultrasound telemedicine service linking a specialist fetal medicine (FM) centre and a remote obstetric unit.

**Methods:**

Over a four-year period from October 2015, all women referred for FM consultation from the obstetric unit were seen via telemedicine, excluding cases where invasive testing, intrauterine therapy or cardiac anomalies were anticipated. The outcomes measured included the indication for FM referral; scan duration and image and sound quality during the consultation. Women’s perceptions of the telemedicine consultation and estimated costs to attend the FM centre were measured by a structured questionnaire completed following the first telemedicine appointment during the Phase 1 of the project.

**Results:**

Overall, 297 women had a telemedicine consultation during Phase 1 (pilot and evaluation) and Phase 2 (embedding and adoption) of the project, which covered a 4 year period 34 women completed questionnaires during the Phase 1 of the study. Travel to the telemedicine consultation took a median (range) time of 20 min (4150), in comparison to an estimated journey of 230 min (120,450) to the FM centre. On average, women would have spent approximately £28 to travel to the FM centre per visit. The overall costs for the woman and her partner/ friend to attend the FM centre was estimated to be £439.

Women were generally satisfied with the service and valued the opportunity to have a FM consultation locally.

**Conclusions:**

We have demonstrated that a fetal ultrasound telemedicine service can be successfully introduced to provide FM ultrasound of sufficient quality to allow fetal diagnosis and specialist consultation with parents. Furthermore, the service is acceptable to parents, has shown a reduction in family costs and journey times.

**Supplementary Information:**

The online version contains supplementary material available at 10.1186/s12884-020-03532-4.

## Background

Ultrasound screening and diagnosis of fetal abnormality and wellbeing is universally offered in the UK and most high income countries, with the aim of providing parents with accurate information to inform timely intervention [1]. In England, ultrasound screening for fetal anomalies is carried out by qualified sonographers during the first trimester and second trimester [1]. The caseload and complexity of pregnancy complications that can be managed locally, in obstetric units, is largely dependent on the availability of health care professionals with the expertise required to interpret fetal ultrasound images and provide appropriate counselling [2]. Women in the UK are often referred from their local obstetric unit to a specialised fetal medicine (FM) centre when a fetal anomaly is suspected, intensive fetal monitoring is required or the mother’s medical or obstetric history indicates that consultation with a fetal medicine specialist is warranted.

Telemedicine can be used to provide healthcare at a distance thereby overcoming challenges relating to geography and the availability of medical expertise. It has previously been used to provide FM consultations [3] and also to undertake real time fetal echocardiography [4], offering women the option to receive prenatal diagnosis and consultation with a specialist, whilst remaining in their local hospital. Providing ultrasound consultations via a telemedicine link has previously been shown to be highly acceptable to women [5]. In addition, telemedicine can be used to provide ultrasound training remotely [5, 6] thereby improving obstetric sonographers’ skills in diagnosing fetal anomalies and monitoring fetal wellbeing and reducing the number of cases referred to FM centres. The opportunity to reduce the need for patients to travel to different hospitals is particularly relevant in the current COVID-19 pandemic situation [7].

For families living in rural areas, access to a FM centre can involve travelling long distances and incurring costs due to loss of earnings, childcare and travel. As an example, women living in parts of Cumbria, UK need to travel up to six hours to attend the FM centre in Newcastle-upon-Tyne for specialist fetal medicine opinion. Cumbria is affected by significant challenges in the provision of health care due to a predominantly rural and isolated population, [8] with some areas affected by high levels of socio-economic deprivation [9]. The combination of these factors means that long journey times are frequently associated with high family costs as well as significant inconvenience for families.

There are recognised challenges to the introduction of telemedicine into routine service provision despite encouraging results from research studies [10–12]. This paper reports the implementation of a successful fetal ultrasound telemedicine service, incorporating an evaluation of women’s views of the service and a comparison of family costs.

## Methods

The project involved two phases:

- **Phase 1** – (12 months, from October 2015 to October 2016) – The pilot and evaluation phase, included establishing the video-conferencing between the FM centre and the obstetric unit, the advanced training of sonographers and collection of participant questionnaire data. Patient experience data was collected throughout the initial 12 month, funded set-up phase (2015–2016).
- **Phase 2** (36 months, from November 2016 to October 2019) – The embedding and adoption phase, which involved increasing the volume and complexity of cases including the introduction of multidisciplinary consultations.

The service was implemented at a specialist FM centre in North East England, and an obstetric unit situated in the North West of England (~ 1200 births per annum) from October 2015. The service utilised existing 2 x 100Mbp/s fibre optic circuits, installed between the hospitals during the pilot phase of the project to support another clinical service. A codec was installed at both sites together with a Cisco Video Conferencing (VC) unit at the FM centre and a Polycom Group 500 VC unit at the obstetric unit to allow the handling of high quality images using the least bandwidth. A bespoke unit including a monitor, microphone, camera and codec was assembled for use in the clinical ultrasound room. The view at the FM centre could be alternated (using a remote control) between the ultrasound machine display during scans, to the woman and her family during pre- and post-scan counselling. The FM consultant and the family had a full screen view of each other when discussing the scan findings.

Information Technology (IT) support was provided by a 24-h help desk at the FM centre and by the local IT team at the obstetric unit. The telemedicine link was available for one session (~ 3 h) per week requiring the provision of an obstetric sonographer. A midwife was present throughout the consultation to provide support to the woman and family at the obstetric unit.

Referrals from the obstetric unit for FM opinion were assessed for telemedicine suitability by one of the FM consultants or the lead FM midwife. All appropriate cases were identified following routine ultrasound screening undertaken at the local unit by sonographers and women were offered telemedicine consultation. Cases were excluded from telemedicine consultation and women offered face-to-face consultations for the following reasons: (a) anticipated need for invasive diagnostic or therapeutic intervention (b) structural cardiac anomaly [due to presence of separate fetal cardiology clinics] (c) suspected facial clefts [due to the need for 3D ultrasound]. Measurement of nuchal translucency (as part of combined testing for common trisomies in twin pregnancies), previously undertaken at the FM centre, was introduced in June 2016 with scans being viewed by an experienced midwife sonographer. An information sheet, as well as verbal explanation of the process was provided to all women prior to the telemedicine consultation. Standard operating procedures were issued to staff, which included guidance on the equipment set-up, patient referral and suitability assessment, consultation process and action in case of link failure.

Three experienced sonographers, each with over 8 years’ obstetric ultrasound experience, completed a training programme during the first four weeks of the project. The sonographers familiarised themselves with the teleconferencing equipment while undertaking fetal growth scans, supported by the team at the FM centre. This provided the opportunity to test the quality and reliability of the transmitted audio and ultrasound images and to undertake training in the acquisition and interpretation of uterine artery (UA) and middle cerebral artery (MCA) Doppler. Sonographers at the obstetric unit did not perform UA or MCA Doppler prior to the implementation of the telemedicine link and women were previously referred to the FM centre if these investigations were indicated. The sonographers were provided with a pre-training manual and remote guidance via the telemedicine link from an experienced midwife sonographer based at the FM centre.

Ultrasound scans at the obstetric unit were performed, using a Toshiba Aplio 400 ultrasound machine. During the ultrasound consultation, one of four FM specialists provided verbal guidance to the sonographer via the telemedicine link to ensure that the necessary images and measurements were obtained. During the scan, the ultrasound image was viewed directly and synchronously by the FM specialist; the image was transmitted from the ultrasound machine at the obstetric unit through a High Definition Multimedia Interface (HDMI) cable to the HDMI port in the codec and viewed on a monitor at the FM Centre. Following the scan, the FM consultant discussed the findings and implications with the woman and her partner/supporting person. A scan report was sent to the referring clinician via a secure email service (NHS Mail) immediately following the telemedicine consultation.

All women undergoing their first telemedicine appointment during Phase 1 of the project were invited to complete a questionnaire following their consultation. The participants were women who had been referred for a FM specialist consultation via telemedicine because of a suspected fetal complication. The aim was to evaluate respondent’s perceptions of the experience of the consultation, including whether they felt involved in their care, knew who to contact with concerns and whether they would choose to use a telemedicine consultation in the future. The questions were based on items used in a previous study [13] and responses were recorded using a five-point Likert scale. Respondents were asked to record the actual costs incurred to attend the telemedicine appointment (for example, travel and childcare costs) and estimated costs of travel and other associated expenses to the FM centre (supplementary file 1). The FM consultants completed a Likert-scale to assess the quality of the image and audio for both the ultrasound scan and subsequent discussion following each consultation. Descriptive analysis of data was performed using SPSS version 21.0 for Windows.

## Results

**Fig. 1:**
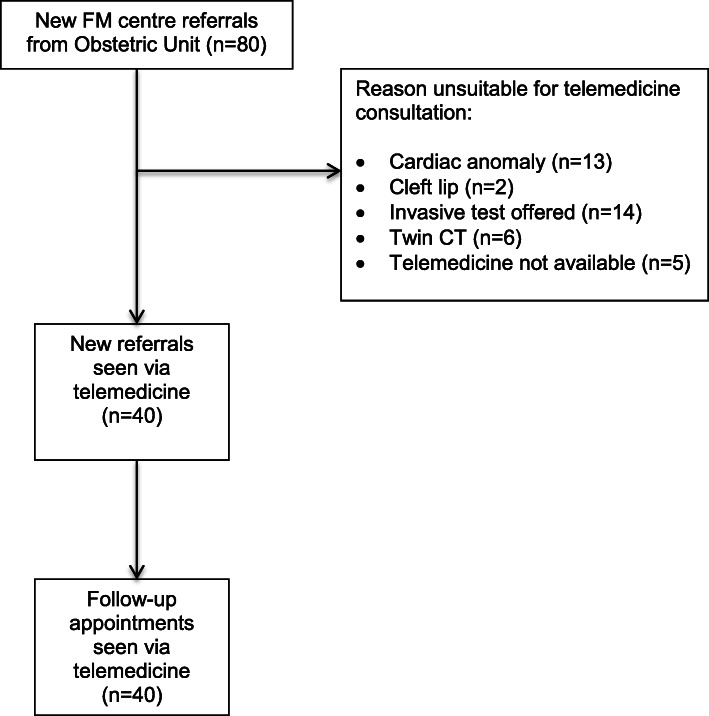
Telemedicine referrals during Phase 1

Figure 1 shows the total number of referrals from the obstetric unit and follow up scans during Phase 1 and the number of those who were unsuitable for a telemedicine consultation.

During the Phase 1 and Phase 2, a total of 297 consultations (ultrasound scan and counselling) were undertaken via the telemedicine link; 154 new and 143 follow-up consultations (Table 1). Scans were most frequently undertaken to assess whether a fetal anomaly was present following referral for FM specialist opinion with renal (*n*=23), central nervous system (*n*=12) and abdomen and gastrointestinal (n=12) anomalies being the most commonly cited reason for referral. A high number of scans were undertaken via telemedicine for intensive monitoring of potentially compromised fetuses, namely small-for-gestational age (*n*=42), alloimmunisation (*n*=21) and fetal infection (*n*=15).

**Table 1:** Indication for new referrals and follow up appointments during Phases 1 and 2

Primary Indication	Sub-group indication	Phase 1 number of new referrals	Phase 1 number of follow-up appointments	Phase 2 number of new referral appointments	Phase 2 number of follow-up appointments
Suspected fetal anomaly	Renal, urinary & genitalia	5	2	18	9
	Central nervous system	6	4	6	5
	Heart arrhythmia	1	0	1	2
	Musculoskeletal	0	0	7	0
	Thorax and diaphragm	0	0	1	8
	Abdominal wall and gastrointestinal	3	4	9	0
	Tumour	0	0	1	2
Small-for-gestational age	Singleton	4	17	8	23
Multiple pregnancy	Twin-to-twin transfusion syndrome screening and management	3	1	1	5
	Small-for-gestational age	0	0	3	2
	Combined testing	2	0	22	0
	Other e.g. single demise	0	0	1	2
Placenta	Abnormally invasive placenta screening and management	11	3	15	4
	Praevia and other (e.g. antepartum haemorrhage)	1	0	10	8
Amniotic fluid	Oligo/anhydramnios	0	0	1	1
	Preterm premature rupture of membranes	1	1	0	0
Fetal infection		0	0	6	15
Combined testing	High risk result	0	0	1	0
Alloimmunisation		1	8	1	13
Previous history	Structural or genetic anomaly	2	0	2	6
	**Total**	**40**	**40**	**114**	**103**

Data from the Phase 1 and Phase 2 showed that the mean duration of telemedicine ultrasound scans was 16 min (SD 7.3). For all scans undertaken via the link the median gestational age at the time of the consultation was 28 weeks 2 days weeks (200 days gestation) (153–230).

During both phases of the project, the imaging and audio quality were rated highly by four FM consultants and one midwife sonographer (twin combined screening only) with an image median rating of 4 out of 5 (4–5) and the audio median rating of 5 out of 5 (5–5). There was only one case (possible abnormally invasive placenta) where the FM consultant was unable to make a definitive prenatal diagnosis due to the quality of the colour Doppler imaging. Based on feedback from the obstetric unit, there were no known cases of incorrect diagnosis during the project duration. During Phases 1 and 2, the link failed to connect three times and disconnected on three other occasions after completion of the ultrasound scan; the post scan consultation was undertaken by telephone.

### Women’s evaluation of the service

Questionnaires were completed by 34/40 (85%) women, following their first telemedicine consultation during Phase 1 of the study. Table 2 shows the demographic characteristics of the respondents.

**Table 2:** Demographic information

Participant characteristics	Survey (%) ***n***=34
**Age**	
16–25	9 (26.6)
26–35	16 (55.9)
35+	6 (17.6)
**Education**	
No formal qualifications	4 (11.8)
GCSE	6 (17.6)
A Level	2 (5.9)
Vocational	14 (41.2)
Undergraduate	6 (17.6)
Postgraduate	2 (5.9)

Overall, women expressed high levels of satisfaction with the telemedicine consultation (Table 3). Only two women returned responses of ‘Strongly Disagree’, one in relation to the quality of the ultrasound image and another to the question of whether she would be willing to use telemedicine to monitor her baby’s health in the future.

**Table 3:** Patient evaluation of telemedicine consultation (Likert scores)

Questionnaire items	Mean Likert score/5 (SD)	% patients agreed or strongly agreed
I was satisfied with the picture quality	4.71 (0.78)	96.7
I was satisfied with the sound quality	4.84 (0.37)	100
I was satisfied with my discussion with the doctor after the scan.	4.90 (0.30)	100
I was able to talk about my concerns openly	4.87 (0.34)	100
I know who to contact with any questions	4.77 (0.50)	97.1
I was involved as much as I wanted to be in decisions about what happens next	4.87 (0.43)	96.7
After the consultation, I have a good understanding of the next steps in my care	4.90 (0.30)	100
I was satisfied with the quality of care received overall	4.90 (0.30)	100
I would be willing to use telemedicine to monitor my baby’s health in the future	4.84 (0.73)	96.7

### Journey times and costs

The majority of women who provided data about costs (*n*=27) [82.3%]), travelled to attend the telemedicine consultation at the obstetric unit by car, the remainder travelling by public transport. The median (range) travel time to the obstetric unit was 20 min (4150) in comparison to an estimated median journey of 230 min (120, 450) to the FM centre.

The additional costs that would have been incurred by respondents if they had travelled to the FM centre included leave from paid employment (*n*=16, 47%), childcare costs (*n*=8, 23.5%) and partner taking time off paid employment (*n*=25, 73.5%). On average, women would have spent approximately £28 to travel to the FM centre per visit. The overall costs for the woman and her partner/ friend to attend the FM centre was estimated to be £439, which included caring responsibilities, and loss of earnings. The link was particularly beneficial to parents requiring regular monitoring (e.g. SGA, alloimmunisation) where multiple journeys to the specialist centre would have otherwise been necessary.

The equipment costs to set-up the service totalled £12,500. Additional salary costs of a sonographer and midwife for 3 h per week to undertake the scans and support women were funded by the obstetric unit. The FM specialist time was not additional to the usual provision of care.

## Discussion

### Summary of results

The project demonstrated that a fetal ultrasound telemedicine service could be successfully implemented in the UK NHS between a remote obstetric unit and a FM centre to provide women with high quality FM consultation and reduce the need for travel. The transfer of ultrasound images is reliable and of sufficiently high quality to achieve fetal diagnosis and consultation in almost all cases.

### Indications for telemedicine

The telemedicine link was effectively utilised to provide ultrasound prenatal diagnosis when a fetal anomaly was suspected and for monitoring the wellbeing of fetuses at risk of compromise, including pregnancies affected by preterm small-for gestational age, preterm premature rupture of membranes, twin-to-twin transfusion syndrome and red cell alloimmunisation.

Women were excluded from a telemedicine consultation if there was a suspected fetal cardiac anomaly because of an existing, separate fetal cardiology service. Previous studies have shown that fetal cardiology by telemedicine is achievable [4, 14, 15] and the telemedicine service could be extended to include such cases in the future. This would require additional training for sonographers and the availability of one of the fetal cardiology team.

It has been previously stated that telemedicine has the potential to increase the number of consultant referrals [16]. It is our experience that sonographers and obstetricians at the obstetric unit are more likely to refer patients for FM review when they are unsure of the diagnosis (rather than repeat a scan themselves). However, the telemedicine link also offers the opportunity for obstetricians and sonographers to discuss uncertainties around ultrasound findings or management of care directly with a specialist consultant, thereby reducing inappropriate referrals. This was also a finding in a previous study of fetal telemedicine [3].

### Benefits to women and acceptability

Women valued the opportunity to receive specialist FM expertise by telemedicine and expressed high levels of satisfaction with the service. These findings are consistent with the findings of previous studies, which showed high levels of patient satisfaction and confidence with telemedicine consultations [3, 5, 13, 17]. The majority of women stated that they would be willing to use the telemedicine service again. Feedback from local staff indicated that some women specifically requested a telemedicine consultation, a finding reported in a previous study [5]. A detailed discussion of the findings from semi-structured interviews undertaken with stakeholders and women who had telemedicine consultations is reported in a separate paper [18].

The value of a fetal ultrasound telemedicine service is highlighted further by the current COVID-19 pandemic [19]; the use of telemedicine reduces the need for face-to face visits while maintaining access to FM consultations, as recommended by current guidance [20]. The pandemic situation has emphasised the opportunities that telemedicine offers when travel needs to be minimised while continuing to provide fetal diagnosis [21].

### Challenges of establishing the service

Difficulties were encountered during the initial stages of the project in establishing a reliable teleconferencing link. Prior attempts to utilise an existing Internet-based video conferencing infrastructure (based on N3 [Wide Area IP Network] and Integrated Services Digital Network (ISDN) telephone lines) were unsuccessful; a connection capable of achieving high quality real-time image transfer was not possible using N3 and direct internet access was unsuccessful as a result of existing firewalls. However, telemedicine consultations can be successfully carried out without the need for dedicated fibre optic connections, meaning that the opportunities for implementation are substantial. Indeed, the fetal telemedicine based at Newcastle upon Tyne Hospitals NHS Foundation Trust has subsequently been expanded to involve more obstetric units utilising a could-based video-conferencing system (StafLeaf).

There were significant challenges for stakeholders responsible for managing staff in the obstetric unit; managers expressed concerns relating to the increased need for sonography and midwifery staff time to support the telemedicine clinic. This was counterbalanced by the opportunity to increase sonographer skill levels and to substantially reduce travel and associated costs for women and families [18]. The initial capital costs associated with the implementation of a telemedicine service are relatively small, albeit they cannot be directly recuperated by delivering remote consultations, meaning that stakeholders have to take this into consideration when planning a service. Furthermore, the introduction of a telemedicine service impacts on the existing workflow and involves reorganisation of staff and clinics [22].

Sonographers expressed worries about their clinical ultrasound practice being observed by the fetal medicine consultant during consultations and being unable to achieve the images required [18]. There was also a need to ensure that there was IT support at both the obstetric unit and FM centre in case of technical problems with the link or equipment, these finding are reported in a separate paper [18]. These concerns were overcome through extended engagement with clinical staff, senior managers and IT managers at both sites for 12 months prior to the start of the project and throughout the embedding phase. The training undertaken with sonographers was fundamental to ensuring that they were confident in their ultrasound skills and use of the telemedicine equipment. Previous studies have shown that successful engagement with clinicians and other stakeholders is a significant factor in the success of telemedicine services, particularly beyond the initial set-up period [12, 23, 24].

### Limitations of study

The relatively small number of referrals from a single, small obstetric unit limits the findings. It is difficult therefore to determine whether the findings are generalisable to other obstetric units and a broader range of clinical specialities. The project was designed to evaluate the implementation and adoption of a fetal telemedicine service but does not provide data on the accuracy of prenatal diagnosis by telemedicine and does not seek to compare telemedicine and non-telemedicine image quality.

## Conclusions

Telemedicine provides a reliable and feasible option for the delivery of FM consultations to women in a small obstetric unit without the need to travel to a FM centre. The service is valued by women and staff and reduces family costs. There is clear scope to develop the use of the telemedicine link within the provision of specialist maternity, neonatal and paediatric care, which would have significant benefits for families, particularly during a pandemic situation. Financial and time saving for patients will vary depending on the distance from the Obstetric Unit to the FM centre. These factors may impact on the capacity to implement telemedicine services across a wider geography.

Future work is focussed on extending the provision to neighbouring obstetric units and to other clinical specialities, particularly in areas where there are challenges to sustaining clinical expertise.

## Supplementary Information

**Additional file 1:.** Participant questionnaire.

## Data Availability

The dataset used in the present study is available from the corresponding author upon reasonable request.

## Abbreviations

AHSN: Academic Health Science Network
FM: Fetal Medicine
MCA: Middle cerebral artery
OSATS: Objective Structured Assessment of Technical Skill
UAD: Uterine Artery Doppler
VC: Video Conferencing
