# Supplementary material for: Implementation of a fetal ultrasound telemedicine service: women’s views and family costs
Source: BMC Pregnancy Childbirth. 2021 Jan 8;21:38. doi: 10.1186/s12884-020-03532-4 (PMC7793392; doi:10.1186/s12884-020-03532-4)
Supplement: Supplementary file 1 — Additional file 1:. Participant questionnaire. [file 12884_2020_3532_MOESM1_ESM.docx]

Thank you for taking the time to look at this questionnaire. The questionnaire asks about your views of the care that you received in relation to your pregnancy. Please answer as many questions as you feel able to.

**Section one:** First, we would like to ask some questions to find out a little bit more about you:

Q1 What is your age in years? _________________________

Q2 Who would you describe as your main source of support at home?

Partner

Parent

Friend

Other (Please state)_______________________________

Q3 Have you ever attended the Fetal Medicine Unit at Royal Victoria Infirmary, Newcastle upon Tyne before?

Q3A During this pregnancy:

Yes No

Q3B During a previous pregnancy:

Yes No Not applicable

**Section Two: evaluation of healthcare received**

Please use the questions below to tell us what you thought of the care that you received at the telemedicine consultation

Q4 Were you given a given a good explanation of what to expect before the telemedicine session began?

Yes No Not sure

**At the telemedicine session (when you spoke to the Fetal Medicine Specialist doctor in Newcastle):**

Strongly Agree Neutral Disagree Strongly Agree Disagree

Q5 I was satisfied with the video picture quality

Q6 I was satisfied with the video sound quality

Q7 I was satisfied with my discussion with the

doctor after the scan.

Q8 I was able to talk about my concerns openly

Q9 I know who to contact with any questions

Q10 I was involved as much as I wanted to be in

decisions about what happens next

Q11 After the consultation, I have a good

understanding of the next steps in my care

Q12 I was satisfied with the quality of care received

overall

Q13 I would be willing to use telemedicine

to monitor my baby’s health in the future

Q14 How much time did your journey take to and from the session at West Cumberland Hospital (not including the scan)?

**mm**m

**hh**

hours minutes

Q15 How much time would your journey to and from a consultation at Newcastle have taken?

**mm**m

**hh**


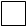


hours minutes Not sure

Q16A If ‘Not sure’, could you please provide your postcode to enable us to work this out? _______________

Q17 Which of the following would apply to you if you had to travel to the Fetal Medicine Unit in Newcastle instead of having a telemedicine consultation? You may tick more than one box.

I would have to take half a day’s leave from paid employment

I would have to take one day’s leave from paid employment

I would be using my maternity leave

I would have to pay childcare costs

Not applicable

Other, please state ________________________________________________________________

Q18 Would your partner or relative/friend have to take time off paid work to have been able to attend the consultation at the RVI with you?

Yes No Not applicable

Q18A If the answer above was yes, would he/she have to miss work for: (choose one if applicable)

½ a day 1 day

Q19 Could you please estimate total loss of earnings and/or other costs e.g. travel?

| Estimated costs | Me | My partner/friend |
| --- | --- | --- |
| Loss of earnings | £ | £ |
| Travel costs | £ | £ |
| Childcare costs | £ | £ |
| Other – please state | £ | £ |
|  | £ | £ |

**Q20 Would your journeys to the RVI have been taken by car or using public transport?**

Car Public transport

Q21 Which of the following best describes **the highest** qualification you have obtained?

No formal qualifications

GCSE level (e.g. GCSE, GCE, O level, Lowers)

A Level (e.g. A level, AS level, Highers)

Vocational qualification (e.g. NVQ, SVQ, BTEC, HND, HNC)

Undergraduate Degree (e.g. BA hons or BSc hons)

Postgraduate degree (e.g. MSc, PhD or Postgraduate professional qualifications)

Other (Please specify)_______________________________

Q21 Please use this space to tell us anything else you would like us to know about your experience of the telemedicine consultation (please use the back page of this questionnaire if you need to):

~~~~~~~~~~~~~~~~~~~~~~~~~~~~~~~~~~~~~~~~~~~~~~~~~~~~~~~~~~~~~~~~~~~~~~~~~~~~~~~~~~~~~~

**In the next stage of the study, we would like to interview some parents about their experience of the telemedicine session over the telephone.**

May we contact you again about this? Yes🞎 No 🞎

If yes, please let us have your name and preferred contact details, and the best time to contact you:

Name: _______________________________________________
